# Influence of processing conditions on apparent viscosity and system parameters during extrusion of distiller's dried grains‐based snacks

**DOI:** 10.1002/fsn3.534

**Published:** 2017-11-27

**Authors:** Poonam Singha, Kasiviswanathan Muthukumarappan, Padmanaban Krishnan

**Affiliations:** ^1^ Department of Agricultural & Biosystems Engineering South Dakota State University Brookings SD USA; ^2^ Department of Dairy and Food Science South Dakota State University Brookings SD USA

**Keywords:** distiller's dried grains, extrusion, garbanzo flour, specific mechanical energy, torque, viscosity

## Abstract

A combination of different levels of distillers dried grains processed for food application (FDDG), garbanzo flour and corn grits were chosen as a source of high‐protein and high‐fiber extruded snacks. A four‐factor central composite rotatable design was adopted to study the effect of FDDG level, moisture content of blends, extrusion temperature, and screw speed on the apparent viscosity, mass flow rate or MFR, torque, and specific mechanical energy or SME during the extrusion process. With increase in the extrusion temperature from 100 to 140°C, apparent viscosity, specific mechanical energy, and torque value decreased. Increase in FDDG level resulted in increase in apparent viscosity, SME and torque. FDDG had no significant effect (p > .5) on mass flow rate. SME also increased with increase in the screw speed which could be due to the higher shear rates at higher screw speeds. Screw speed and moisture content had significant negative effect (*p *<* *.05) on the torque. The apparent viscosity of dough inside the extruder and the system parameters were affected by the processing conditions. This study will be useful for control of extrusion process of blends containing these ingredients for the development of high‐protein high‐fiber extruded snacks.

## 1 INTRODUCTION

Extrusion cooking is a popular food processing technique that has been extensively used to produce protein‐rich and fiber‐rich products. Extrusion process is a combination of cooking, mixing and forming, resulting in good quality direct expanded product. It is widely used in industries owing to its characteristic high throughput and automatic control (Singh & Muthukumarappan, 2014b). During extrusion, the interactive effects of temperature, shearing forces, and moisture content of the blend transform the feed ingredients at macroscopic and microscopic levels leading to structural changes of protein and starch (Brown, Fallahi, Muthukumarappan, Singha, & Sindelar, 2015). Starch is gelatinized, protein is denatured and enzymes, microbes and many anti‐nutritional factors are inactivated.

The extent of mixing, shearing and compressing of the materials, and the rate of heating inside the extruder and die, depends on the raw materials and process conditions used (Singh & Muthukumarappan, 2015). The extrusion process also depends on the pressure developed inside the die and the degree to which the screw is filled (Singh & Muthukumarappan, 2014a). Hence, it is important to understand the rheological changes encountered by an ingredient inside the barrel. Each material that flows inside the extruder has its own properties and behaves differently. The behavior can be quantified by determining mass flow and energy responses. Apparent viscosity is one of the most important rheological properties which has a direct impact on the quality of a final product. A continuous monitoring system is often utilized to measure the apparent viscosity during the extrusion process (Chen, Jao, Larkin, & Goldstein, 1978; Lam & Flores, 2003). It is a good indicator of dough's behavior and the changes during processing. Protein denaturation and polysaccharide gel formation can affect viscosity during extrusion (Bhattacharya & Hanna, 1986). Separate attachments have been employed to measure the dough rheology of wheat, corn, and soybeans using straight tube viscometers (Harper, Rhodes, & Wanniger, 1971), cylindrical dies of different lengths (Harmann & Harper, 1974), capillary die rheometers (Singh & Muthukumarappan, 2016a,b), and viscoamylographs (Remsen & Clark, 1978) attached to food extruders. Not only the feed material but the quality of extrudates depends on the type of extruder used, choice of screw configuration, moisture content of the feed, temperature profile in the barrel and die, and screw speed (Singha & Muthukumarappan, 2016).

Dry‐milling process involved in corn ethanol production, produces distillers dried grains (DDG) and distillers dried solubles (DDS). Distillers dried grains with solubles (DDGS) is produced after mixing and drying these two co‐products (Singh, 2016). DDGS contain high levels of protein since most of the starch is removed (Rosentrater & Krishnan, 2006). It is usually used as cattle feed. However, few studies have been reported on its application in human food (Rasco & McBurney, 1989; Rosentrater & Krishnan, 2006; Wu, Youngs, Warner, & Bookwalter, 1987). The growing interest in the health benefit of protein and fiber justifies exploring the use of DDGS as a protein and fiber supplement in food products. DDGS supplementation will improve the nutritive value of food products by enriching their protein and fiber content, and expand the use of the co‐product from alcohol fermentation (Tsen, Eyestone, & Weber, 1982). DDG and DDGS have been extensively used as a protein source for the development of pet food and aquafeed. Few studies have also been reported on the addition of DDG in snack foods. Reddy and Stoker (1993) added DDGS in wheat flour for the preparation of noodles and baked foods.

Cereal or grains are the primary source of most extruded snack foods because of their high expansion properties. However, they tend to be low in protein and essential nutrients. Usually, cereals lack lysine as the essential amino acid but have sufficient sulfur containing amino acids. Legumes on the other hand, are rich in lysine and deficient in sulfur containing amino acids. When combined together, the proteins of cereals and legumes complement one another to produce a protein of a better quality. Corn in different form has been widely used as raw material for extrusion. It is ideal for extrusion since it has a high starch content, which facilitates the expansion process. It is gluten‐free and contains protein, fiber, vitamins, and unsaturated fatty acids. Compared to wheat, oat, and rice, corn has higher phytochemical content such as phenolic compounds which have anticarcinogenic effects (Jideani et al., 2014). Pulse crops (garbanzo, lentils, dry beans, lupin, and various types of beans) are excellent source of protein, complex carbohydrates, fibers, essential vitamins and minerals. Additionally, they are low in fat and sodium content, no cholesterol, and high in phenolics and bioactive compounds (Roy, Boye, & Simpson, 2010). Inclusion of pulses and legumes increases the protein content and improves potential nutritional content owing to increase in protein digestibility. This was reported by Tiwari, Brennan, Jaganmohan, Surabi, and Alagusundaram (2011) while studying the addition of pigeon pea to wheat flour‐based biscuits and, de la Hera, Ruiz‐París, Oliete, and Gómez (2012) while studying the effects of addition of legume flour to traditional cereal‐based flours. Madhumitha and Prabhasankar (2011) reported that there was an improvement in the nutritional value of pasta by adding black gram flour and mentioned that the processing of food material increases the value and shelf life of the product. Extrusion treatment of lentil flours has also been linked with increase in some bioactive components (Morales et al., 2015).

Garbanzo flour contain moderately high protein (17%–22%), low fat (6.48%), high available carbohydrate (50%) and crude fiber contents of 3.82% (Alajaji & El‐Adawy, 2006). Garbanzo has significant amounts of calcium, potassium, phosphorus, zinc, magnesium, and iron. Garbanzo is known to reduce cholesterol and blood glucose levels (Singh & Singh, 1992). They are increasingly used in healthy diets to promote general well‐being and to reduce the risks of cardiovascular diseases and diabetes. Development of garbanzo‐based snacks could provide enhanced uses for chickpea. Processing of garbanzo into extruded snacks is limited (Bhattacharya & Prakash, 1994; Meng, Threinen, Hansen, & Driedger, 2010; Shirani & Ganesharanee, 2009). Little information is available on the effect of extrusion on system parameters using garbanzo as one of the ingredients (Meng et al., 2010).

Blending of DDGS and garbanzo flour will serve as a good raw material for gluten‐free healthy alternative snacks for a healthy conscious population. For our study, DDGS was processed for food application and was named FDDG. We blended garbanzo flour, FDDG and corn grits at different levels. Our objective was to study the effect of the feed moisture content, screw speed, and barrel and die temperature, on the apparent viscosity and the system parameters (specific mechanical energy, torque, and mass flow rate) during extrusion cooking.

## 2 MATERIAL AND METHODS

### 2.1 Raw materials and blend preparation

Distiller's dried grains with solubles (DDGS) was obtained from Glacial Lakes Energy LLC, Watertown, SD. It was then washed, freeze dried, steam/pressure sterilized, oven toasted, and ground to make a wholesome food‐grade ingredient. The processing of DDGS for food application was done following method described by Rosentrater and Krishnan (2006). The DDGS processed specifically for food application studies is referred to as FDDG henceforth. The initial moisture content of FDDG was 0.70%. The proximate composition of FDDG was: 35.12% protein, 0.53% fat, 1.24% ash, 35.00% dietary fiber, and 13.03% nitrogen‐free extract (dry basis). The FDDG was stored at −20°C until further use. Garbanzo flour (GF) was purchased from a local store in, Brookings, SD. The initial moisture content of GF was 10.08%. The proximate composition of the GF was: 22.42% protein, 5.94% fat, 2.70% ash, 6.56% fiber, and 54.63% nitrogen‐free extract (dry basis). Corn grits (CG) was obtained from Bob's Red Mill (Milwaukie, OR). The initial moisture content of CG was 11.46%. The proximate composition of the CG was: 6.00% protein, 1.50% fat, 2.00% ash, 0.90% fiber, and 78.14% nitrogen‐free extract (dry basis). The different ingredients, that is, FDDG, GF and CG were mixed into five different compositions (Blend I–V) as shown in Table 1. Water was added to the blends to make 14%–20% (wet basis) final moisture depending on the experimental runs (Table 2). The ingredients were mixed in a laboratory scale mixer (KitchenAid Professional 5 Plus, Troy, Ohio, USA) for 10 min. For moisture stabilization, the blends were stored overnight at ambient temperature. The moisture content of the prepared blends was determined using the method 44–19 (AACC, 2000). The proximate composition of blends is shown in Table 1.

**Table 1:** Ingredient composition of blends

Feed ingredients	Percentage of ingredients (% db)				
	Blend I	Blend II	Blend III	Blend IV	Blend V
FDDG	0	5	10	15	20
Garbanzo flour	40	35	30	25	20
Corn grits	60	60	60	60	60
Proximate analysis					
Protein (% db)	13.79	15.06	16.34	17.62	18.90
Fiber (% db)	3.45	4.86	6.27	7.67	9.08
Fat (% db)	3.59	3.30	3.00	2.71	2.41
Ash (% db)	2.53	2.44	2.36	2.27	2.19
NFE (% db)	76.64	74.34	72.03	69.73	67.42

FDDG = Distiller’s Dried grains processed for food application, db = dry basis.

**Table 2:** Independent numerical variables and their levels

Numerical variable	Symbol	Coded variable levels				
		−2	−1	0	1	2
FDDG (%)	*X*_1_	0	5	10	15	20
Temperature (°C)	*X*_2_	100	110	120	130	140
Screw speed (rpm)	*X*_3_	100	125	150	175	200
Moisture content (% wb)	*X*_4_	14	15.5	17	18.5	20

*wb = wet basis*.

### 2.2 Extrusion processing

**Figure 1:**
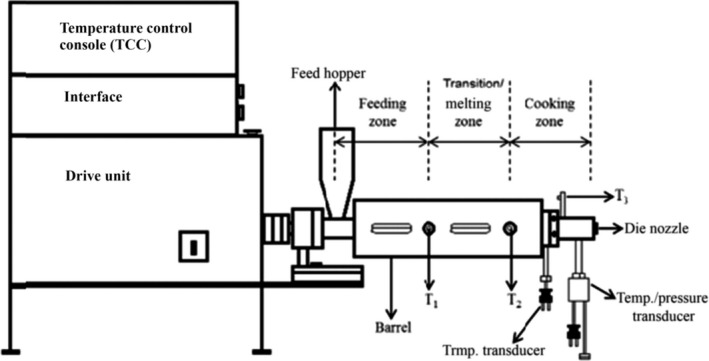
Schematic diagram of a single screw extruder (Source: Singh and Muthukumarappan (2014b))

Post conditioning, the blends were randomly extruded using single‐screw laboratory extruder (Brabender Intelli‐Torque Plasti‐Corder^®^, South Hackensack, NJ) having barrel inner diameter of 19.18 mm. A screw compression ratio of 1.5:1 was used in the experiments. A pictorial representation of the single screw extruder is shown in Figure 1 (Singh & Muthukumarappan, 2014b). Pressure at the die, and net torque exerted on the extruder drive (N‐m) were measured. Stock thermocouples (model 05‐00‐317, C. W. Brabender) were inserted into the barrel and die to measure the dough temperature. Extrudate samples were collected every 30 s to determine the mass flow rate (g/s) by the method described by Rosentrater, Richard, Bern, and Flores (2005). SME (W‐h/kg) consumption was calculated (Lam & Flores, 2003) as:

(1) $$\text{SME}=\frac{\Omega \cdot \omega }{\text{MFR}\left[\frac{3600}{1000}\right]}$$

where Ω is the net torque exerted on the extruder drive (N‐m), ω is the angular velocity of the screw (rad/s) and MFR is the mass flow rate of dough (mass throughput, g/s).

**Figure 2:**
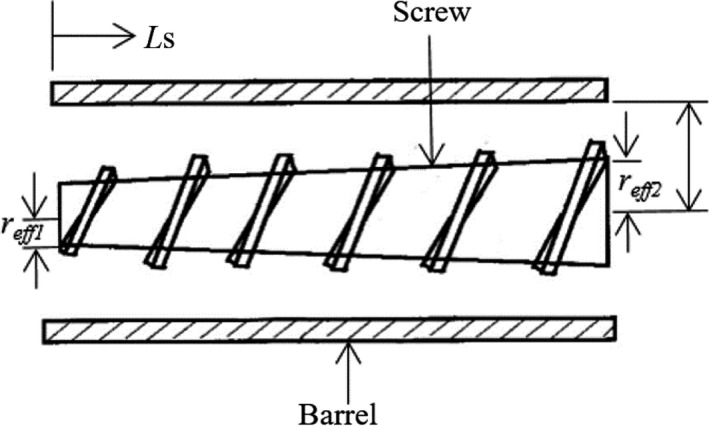
Schematic diagram of a section of single screw (Source: Singha and Muthukumarappan (2016))

The apparent viscosity of the dough in the extruder was calculated by approximating extruder behavior as that of a coaxial viscometer but corrected for the tapered screw geometry (Figure 2) of the extruder barrel (Konkoly, 1997; Lam, 1996; Rogers, 1970). As discussed by Lam and Flores (2003), the shear stress (τ_*s*_) at the screw surface (N/m^2^) and the shear rate (${\dot{\gamma }}_{s}$, 1/*s*) were calculated from the following equations:

(2) $$\tau_{s}=\Omega /\left(2\cdot \pi \cdot \left(r_{\mathrm{coor}}\right){}^{2}\cdot L_{s}\right)=C_{\mathrm{ss}}\Omega$$

(3) $${\dot{\gamma }}_{s}=\left(2\cdot \omega \cdot r_{b}^{2}\right)/\left(r_{b}^{2}-\left(r_{\mathrm{coor}}^{2}\right)\right)=C_{\mathrm{sr}}\omega$$

where *r*_*cor*r_ is the radius correction due to the screw's frustum geometry

(4) $$r_{\mathrm{coor}}=\left(\sqrt{(r_{\mathrm{eff}1}^{2}+r_{\mathrm{eff}1}r_{\mathrm{eff}2}+r_{\mathrm{eff}2}^{2})/3}\right)$$

*r*_*eff*_ is the effective radius (m), Ω is the net torque exerted on the screw (N m), *L*_*s*_ is the screw length in the axial direction (m), ω is the angular velocity of the screw (rad/s), *C*_ss_ is an empirical correction factor for shear stress (which is 10321.5 for this study), ${\dot{\gamma }}_{s}$ is the shear rate at the screw surface (1/*s*), *r*_b_ is the inner barrel radius (m), and *C*_sr_ is the empirical correction factor for shear rate (which is 3.48 for this study). The calibration value for this extruder has been calculated from the calculation reported elsewhere (Lam & Flores, 2003). The apparent viscosity was calculated by taking the ratio of Equations (2) and (3).

(5) $$\eta_{\mathrm{app}}=\frac{\tau_{s}}{{\dot{\gamma }}_{s}}=\left(\frac{C_{\mathrm{ss}}}{C_{\mathrm{sr}}}\right)\left(\frac{\Omega }{\omega }\right)$$

where η_app_ is the apparent viscosity of the dough in the extruder (Pa.s).

### 2.3 Experimental design and statistical analysis

Experiments were conducted using the central composite rotatable design which was developed using Design‐Expert 8.0.7.1 (Statease, Minneapolis, MN, USA). Four numerical independent variables namely FDDG (*X*_1_), temperature (*X*_2_), screw speed (*X*_3_), and moisture content (*X*_4_) each at five levels were taken as shown in Table 2. Three replicates were taken (optional) at the design center (0, 0, 0) and the total number of observations were 27 [24 (axial points) and 3 (center points)].

The experimental design and the codes for the processing variables are shown in Table 3.

**Table 3:** Experimental design layout

Run	Coded variable				Actual variable			
	*x*_1_	*x*_2_	*x*_3_	*x*_4_	*X*_1_ (%)	*X*_2_ (°C)	*X*_3_ (rpm)	*X*_4_ (% wb)
1	−1	−1	1	1	5	110	175	18.5
2	1	1	1	1	15	130	175	18.5
3	0	0	2	0	10	120	200	17
4	1	−1	1	1	15	110	175	18.5
5	−1	−1	−1	1	5	110	125	18.5
6	0	2	0	0	10	140	150	17
7	−1	−1	−1	−1	5	110	125	15.5
8	1	−1	−1	−1	15	110	125	15.5
9	2	0	0	0	20	120	150	17
10	0	0	0	−2	10	120	150	14
11	1	1	−1	1	15	130	125	18.5
12	0	0	−2	0	10	120	100	17
13	−1	1	1	−1	5	130	175	15.5
14	0	0	0	2	10	120	150	20
15	0	0	0	0	10	120	150	17
16	1	1	−1	−1	15	130	125	15.5
17	0	0	0	0	10	120	150	17
18	0	−2	0	0	10	100	150	17
19	−1	1	−1	1	5	130	125	18.5
20	1	−1	−1	1	15	110	125	18.5
21	−1	−1	1	−1	5	110	175	15.5
22	−2	0	0	0	0	120	150	17
23	1	−1	1	−1	15	110	175	15.5
24	−1	1	1	1	5	130	175	18.5
25	−1	1	−1	−1	5	130	125	15.5
26	1	1	1	−1	15	130	175	15.5
27	0	0	0	0	10	120	150	17

*wb = wet basis*.

Mass flow rate (*Y*_MFR_), specific mechanical energy (*Y*_SME_), apparent viscosity of dough (*Y*_AV_), and torque (*Y*_Tor_) were taken as the responses. Second‐order polynomial regression models were established for the dependent variables to fit experimental data for each response.

(6) $$y_{i}=b_{0}+\sum_{i=1}^{a}b_{i}x_{i}+\sum_{i=1}^{a}b_{\mathrm{ii}}x_{i^{2}}+\sum_{i=1}^{a}\sum_{j=1}^{a}b_{\mathrm{ij}}x_{i}x_{j}$$

where *y*_i_ is the predicted response; *b*_0_ is the interception coefficient; *b*_i_, *b*_ii_, and *b*_ij_ are coefficients of the linear, quadratic, and interaction terms; and *x*_i_ is the independent variables studied. The fitness of the model was evaluated and the interactions between the independent and dependent variables were identified using an analysis of variance (ANOVA) presented in Tables 5 and 6. The goodness of fit of the second‐order equation was expressed by the coefficient of determination (*R* ^2^) and its statistical significance was determined by the *F*‐test. Three‐dimensional response surfaces were used to visualize the interactive effects of the independent variables.

## 3 RESULTS AND DISCUSSION

### 3.1 Effect of processing conditions on apparent viscosity

The fitted model shown in Table 4 had a significant coefficient of determination (*R* ^2^) of 0.82. The second order model (Table 5) for apparent viscosity was significant (*p *<* *.05), whereas lack‐of‐fit was not significant (*p *>* *.05). The selected model adequately represented the data for apparent viscosity. FDDG (*x*_1_) had significant (*p *<* *.05) positive linear effect, whereas temperature (*x*_2_) and screw speed (*x*_3_) had significant negative linear effects (*p *<* *.05) on apparent viscosity of the dough inside the extruder. Temperature also showed significant negative quadratic effect (*p *<* *.05) suggesting that viscosity decreased with excessive increase in temperature.

**Table 4:** Best‐fit response surface models after excluding the insignificant terms for apparent viscosity (AV), mass flow rate (MFR), specific mechanical energy (SME) and torque (Tor)

Response surface model	*R* ^2^	Adj *R* ^2^
$Y_{\mathrm{AV}}=3020.38+371.45x_{1}-239.75x_{2}-430.65x_{3}-339.85x_{2}^{2}$	0.82	0.61
$Y_{\mathrm{MFR}}=1.93+0.04x_{4}+0.06x_{2}x_{3}-0.05x_{3}x_{4}+0.04x_{3}^{2}$	0.81	0.58
$Y_{\mathrm{SME}}=134.54+7.24x_{1}-8.24x_{2}+11.13x_{3}-11.09x_{4}-13.03x_{2}^{2}$	0.89	0.76
$Y_{\mathrm{Tor}}=15.6+0.77x_{1}-1.47x_{2}-1.25x_{3}-1.33x_{4}-0.76x_{1}x_{3}-1.17x_{2}x_{4}-1.47x_{2}^{2}$	0.95	0.89

**Table 5:** Analysis of variance for apparent viscosity and MFR

Source	*df*	Apparent viscosity				MFR			
		SS	MS	*F*‐value	*p*‐Value	SS	MS	*F*‐value	*p*‐Value
Model	14	15343599.13	1095971.37	3.9220	0.0114	0.2220	0.0159	3.5378	0.0172
*X*_1_‐FDDG	1	3311400.79	3311400.79	11.8501	0.0049	0.0006	0.0006	0.1274	0.7274
*X*_2_‐Temperature	1	1379553.43	1379553.43	4.9368	0.0463	0.0005	0.0005	0.1006	0.7566
*X*_3_‐Screw speed	1	4450944.93	4450944.93	15.9280	0.0018	0.0172	0.0172	3.8378	0.0738
*X*_4_‐Moisture	1	1091398.09	1091398.09	3.9056	0.0716	0.0312	0.0312	6.9675	0.0216
*X*_1_ *X*_2_	1	367469.23	367469.23	1.3150	0.2738	0.0205	0.0205	4.5783	0.0536
*X*_1_ *X*_3_	1	172289.06	172289.06	0.6165	0.4476	0.0003	0.0003	0.0610	0.809
*X*_1_ *X*_4_	1	47959.95	47959.95	0.1716	0.6860	0.0018	0.0018	0.3959	0.541
*X*_2_ *X*_3_	1	203505.23	203505.23	0.7283	0.4102	0.0580	0.0580	12.9321	0.0037
*X*_2_ *X*_4_	1	4701.64	4701.64	0.0168	0.8989	0.0082	0.0082	1.8219	0.2020
*X*_3_ *X*_4_	1	135458.33	135458.33	0.4847	0.4995	0.0387	0.0387	8.6264	0.0124
$X_{1}^{2}$	1	413947.77	413947.77	1.4813	0.2470	0.0081	0.0081	1.8135	0.2030
$X_{2}^{2}$	1	2463928.70	2463928.70	8.8173	0.0117	0.0138	0.0138	3.0719	0.1051
$X_{3}^{2}$	1	18756.19	18756.19	0.0671	0.8000	0.0390	0.0390	8.6929	0.0122
$X_{4}^{2}$	1	817.44	817.44	0.0029	0.9578	0.0170	0.0170	3.8039	0.0749
Residual	12	3353294.49	279441.21	–	–	0.0538	0.0045	–	–
Lack of fit	10	2537336.80	253733.68	0.6219	0.7520	0.0310	0.0031	0.2719	0.9365
Pure error	2	815957.69	407978.84	–	–	0.0228	0.0114	–	–

**Figure 3:**
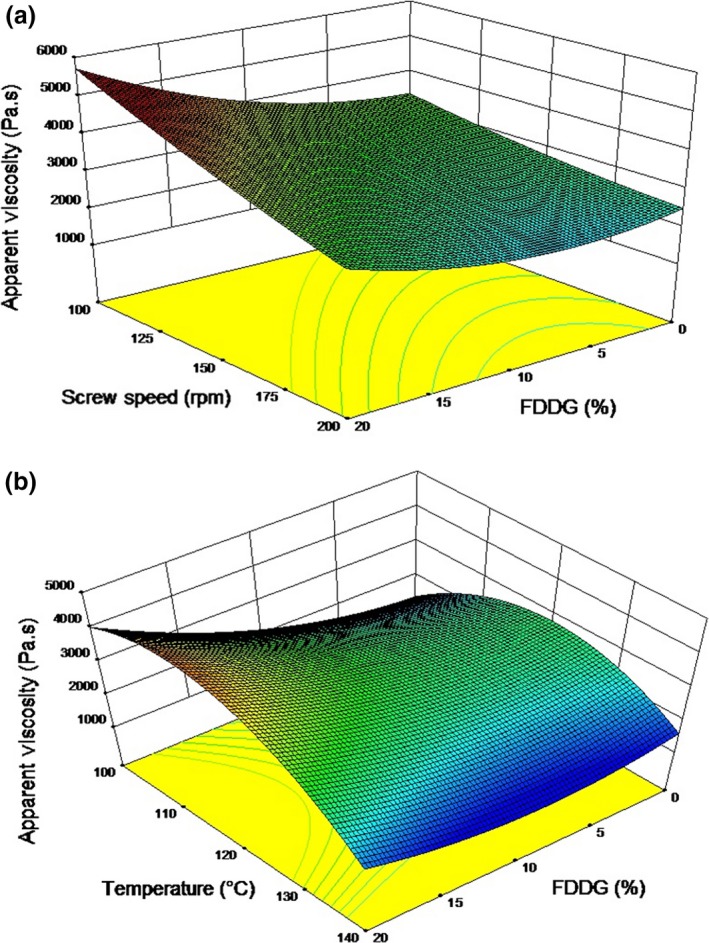
Response surface plots for apparent viscosity as a function of (a) Screw speed and FDDG at 120°C temperature and 17% moisture content; (b) Temperature and FFDG at 150 rpm screw speed and 17% moisture content

The apparent viscosity varied between 1,654 and 4,397 Pas. Although in this study, moisture content did not have any significant effect (*p *>* *.05) on apparent viscosity, the highest viscosity was observed at the lowest moisture content of the blend which was 14%. Response surface plots for apparent viscosity at different FDDG level, temperature and screw speed are shown in Figures 3a and 3b. The apparent viscosity of the dough increased with higher levels of FDDG. Increasing the percentage of FDDG in the blend also increased the protein content. The rise in FDDG content changed the overall chemical composition and the dough functionality which may have resulted in the higher apparent viscosity. Bhattacharya and Hanna (1986) also reported an increase in viscosity when the percentage of soy protein content in the blend was increased during extrusion of corn‐soy mix.

Temperature had a significant quadratic effect (*p *<* *.05) on the apparent viscosity (Figure 3b). It was observed that the apparent viscosity of the dough decreased when temperature was increased and at very high temperature there was a sharp decrease in apparent viscosity. The apparent viscosity decreased with increase in the screw speed from 100 rpm to 200 rpm (Figure 3), indicating shear thinning behavior of the dough (Singh & Muthukumarappan, 2017). This happens due to increased shear rates and molecular degradation (Singh & Muthukumarappan, 2016b). Similar observations were also reported by Chinnaswamy and Hanna (1990).

### 3.2 Effect of processing conditions on mass flow rate

The drag flow developed by screw rotation inside extruder and the pressure developed due to constriction at the die influences the mass flow rate (Ludewig, 1989). Multiple regression equation for MFR (*Y*_MFR_) in terms of coded variables is shown in Table 4. ANOVA for the model of MFR as fitted (Table 5) shows that the model was significant (*p *<* *.05), whereas lack of fit was not significant (*p *>* *.05). The response surface regression model on MFR yielded a good fit with a coefficient of determination (*R* ^2^ = 0.81) for the extrudates. Regression analyses showed that MFR was significantly (*p *<* *.05) affected by linear effect of moisture content (*x*_1_) and quadratic effect of screw speed (*x*_3_). Interaction effects of temperature and screw speed (*x*_2_ *x*_3_), and screw speed and moisture content (*x*_3_ *x*_4_) were also observed.

**Figure 4:**
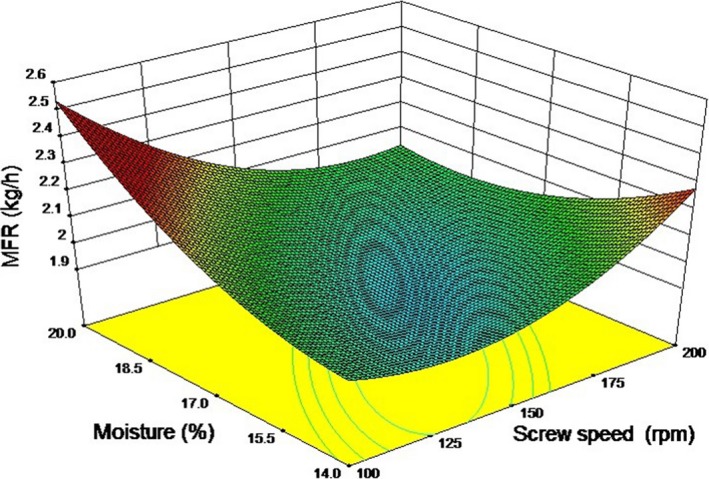
Response surface for mass flow rate as a function of screw speed and moisture content at 120°C temperature and 10% FDDG level

The MFR varied between 1.67 and 2.28 kg/h. The effects of screw speed and moisture content on MFR are shown in Figure 4. Increasing the screw speed from 100 to 200 rpm significantly (*p *<* *.05) increased the MFR. Such behavior is expected as there is a proportional relationship between drag flow in extruder and the screw speed. For this reason, higher screw speeds means higher mass flow rate and there is greater ability for the material to move along the extruder barrel (Harper, 1981). Increasing the moisture content from 14% to 20% also significantly (*p *<* *.05) increased the MFR. High moisture content aides in gelatinization of the dough and thus apparent viscosity decreases which has been observed in this study. This explains the increase in MFR with increase in moisture content of the dough. No significant effect (p>.05) of FDDG on the MFR was observed.

### 3.3 Effect of processing conditions on specific mechanical energy

A multiple linear regression equation of SME (*Y*_SME_) in terms of coded levels is shown in Table 4. Linear terms of FDDG (*x*_1_), temperature (*x*_2_), screw speed (*x*_3_), and moisture content (*x*_4_) had significant effects (*p* < .05) on SME. Temperature had a significant negative quadratic effect (*p* < .05) on SME. SME increased with increase in temperature, while excessive increase in temperature resulted in decrease in SME. The fitted quadratic model had a coefficient of determination (*R* ^2^) of 0.89. The model (Table 6) for SME was significant (*p *<* *.05), whereas lack of fit was not significant (*p *>* *.05).

**Table 6:** Analysis of variance for SME and torque

Source	*df*	SME				Torque			
		SS	MS	*F*‐value	*p*‐Value	SS	MS	*F*‐value	*p*‐Value
Model	14	15402.4562	1100.1754	6.7475	0.0010	254.0629	18.1473	16.1637	<0.0001
*X*_1_‐FDDG	1	1258.3786	1258.3786	7.7178	0.0167	14.0558	14.0558	12.5194	0.0041
*X*_2_‐Temperature	1	1629.9350	1629.9350	9.9966	0.0082	51.8246	51.8246	46.1598	<0.0001
*X*_3_‐Screw speed	1	2972.2487	2972.2487	18.2291	0.0011	37.4933	37.4933	33.3951	<0.0001
*X*_4_‐Moisture	1	5468.2265	5468.2265	33.5372	<0.0001	42.4260	42.4260	37.7886	<0.0001
*X*_1_ *X*_2_	1	4.2068	4.2068	0.0258	0.8751	3.0900	3.0900	2.7523	0.1230
*X*_1_ *X*_3_	1	35.6478	35.6478	0.2186	0.6485	9.1869	9.1869	8.1828	0.0143
*X*_1_ *X*_4_	1	10.9873	10.9873	0.0674	0.7996	0.6546	0.6546	0.5831	0.4599
*X*_2_ *X*_3_	1	228.0132	228.0132	1.3984	0.2599	2.5868	2.5868	2.3041	0.1549
*X*_2_ *X*_4_	1	45.8293	45.8293	0.2811	0.6057	22.0873	22.0873	19.6730	0.0008
*X*_3_ *X*_4_	1	16.2969	16.2969	0.1000	0.7573	0.1693	0.1693	0.1508	0.7046
$X_{1}^{2}$	1	147.8367	147.8367	0.9067	0.3598	1.6217	1.6217	1.4444	0.2526
$X_{2}^{2}$	1	3621.7322	3621.7322	22.2125	0.0005	46.2688	46.2688	41.2113	<0.0001
$X_{3}^{2}$	1	538.2015	538.2015	3.3008	0.0943	0.1660	0.1660	0.1478	0.7073
$X_{4}^{2}$	1	268.1656	268.1656	1.6447	0.2239	1.4779	1.4779	1.3164	0.2736
Residual	12	1956.5932	163.0494	–	–	13.4726	1.1227	–	–
Lack of fit	10	1834.2774	183.4277	2.9992	0.2759	9.6126	0.9613	0.4981	0.8151
Pure error	2	122.3158	61.1579	–	–	3.8600	1.9300	–	–

**Figure 5:**
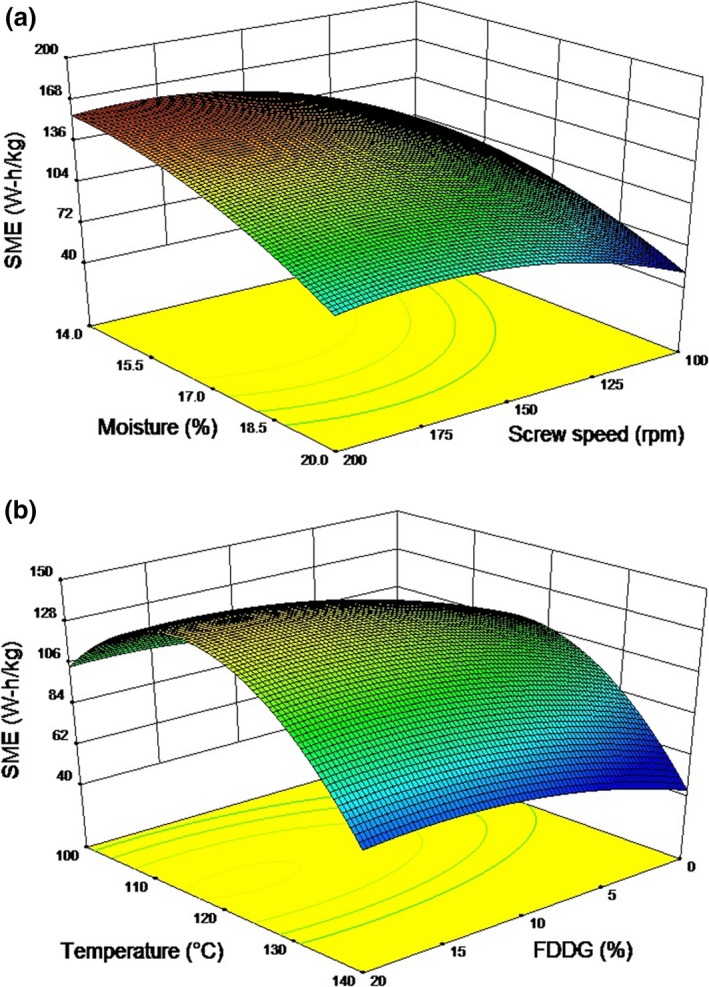
Response surface plots for specific mechanical energy as a function of (a) Moisture content and screw speed at 120°C and 10% FDDG level, (b) Temperature and FDDG at 150 rpm screw speed and 17% moisture content

The amount of mechanical energy input during extrusion has a direct role in macromolecular transformations and interactions of different components in the feed materials. The SME in this study varied from 61 to 164 W‐h/kg. Figures 5a and b shows the response surface graph of SME versus moisture content and screw speed, and FDDG level and temperature, respectively. SME increased with increase in FDDG level. This could be due to reduced starch content in the dough containing higher FDDG. Furthermore, we have observed previously that viscosity increased with increase in FDDG. This indicates that higher energy and pressure is required during the extrusion of the blends containing higher percent of FDDG. The high fiber content has a tendency to bind more water, resulting in reduced availability of water for starch (Mir, Bosco, Shah, Santhalakshmy, & Mir, 2015). SME increased initially with increase in temperature but further increase in temperature resulted in decrease in SME. High temperatures are normally associated with a decrease in the melt viscosity inside the extruder, which in turn reduces the energy input of the extruder. According to Ludewig (1989), with increase in screw speed, the SME generally increases. This is because the magnitude of change in energy input to the screw is typically greater than the decrease in torque associated with the decrease in apparent viscosity due to shear thinning behavior of the non‐Newtonian materials. The high SME at high screw speed and low barrel temperature was also observed by Meng et al. (2010) during extrusion of chickpea and whey protein‐based blends. Increase in feed moisture during extrusion is associated with decrease in viscosity which ultimately leads to reduced SME (Chang, Martinez‐Bustos, Park, & Kokini, 1999; Hsieh, Huff, Lue, & Stringer, 1991). Such findings are in agreement with Bhattacharya and Hanna (1987) and Filli, Nkama, Jideani, and Ibok (2012).

### 3.4 Effect of processing conditions on torque

The multiple regression equation for torque (*Y*_Tor_) in terms of coded variable is given in Table 4. The torque was influenced significantly (*p *<* *.05) by negative linear effects of FDDG (*x*_1_), temperature (*x*_2_), screw speed (*x*_3_), and moisture content (*x*_4_) suggesting that increase in the levels of these variables resulted in decrease in torque. Temperature also had significant negative quadratic effect (*p *<* *.05) on torque indicating excessive increase in temperature reduced the torque of the extruder. Interaction effects of FDDG and screw speed (*x*_1_ *x*_3_) and temperature and moisture content *(x*_2_ *x*_4_) were also observed. The responses were analyzed using ANOVA and the data are presented in Table 6. Examination of the model shows a good fit with *R* ^2^ equal to 0.95 for the torque. The linear model was significant (*p *<* *.05), whereas lack of fit was not significant (*p *>* *.05) for torque.

**Figure 6:**
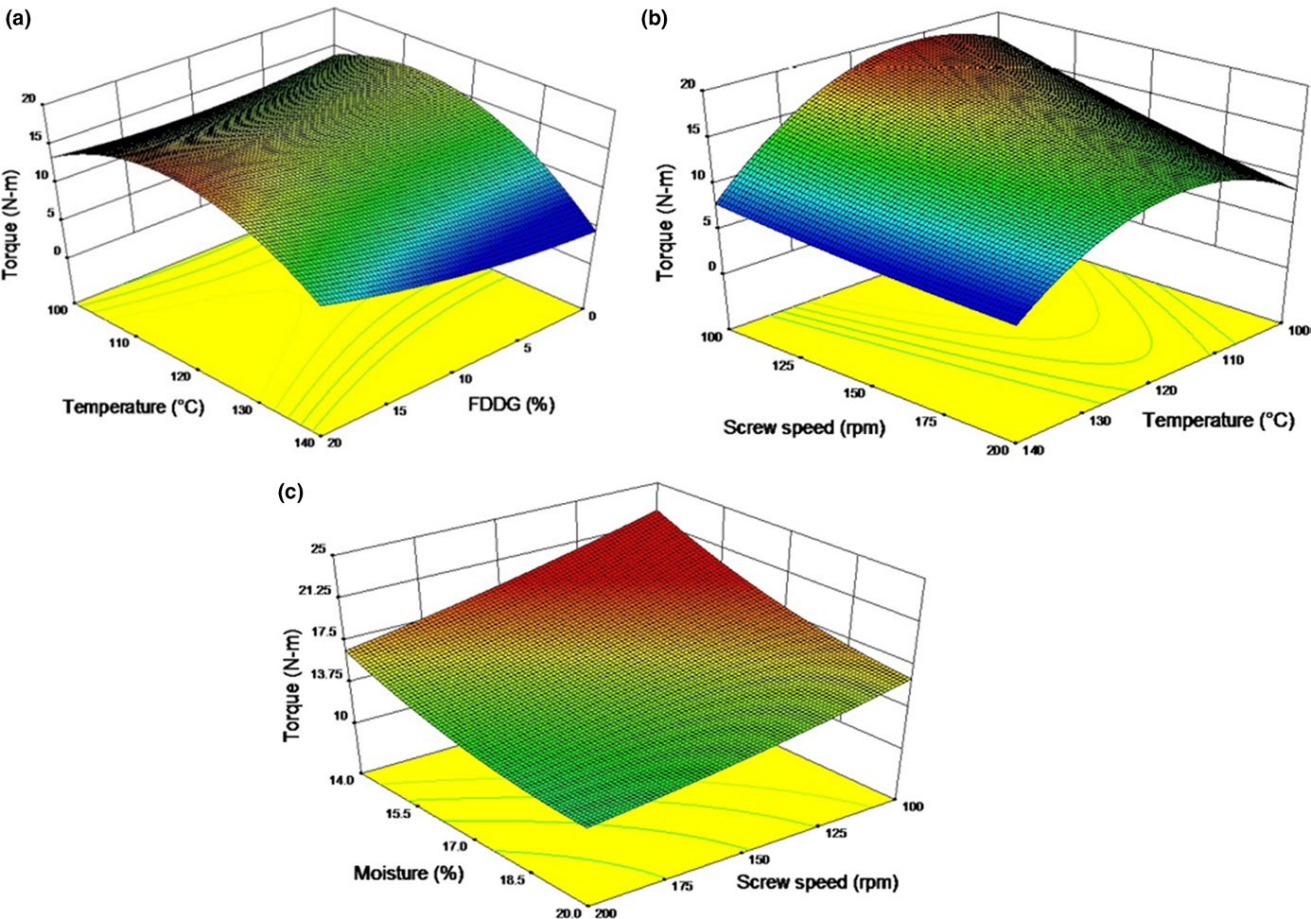
Response surface plots for torque as a function of (a) FDDG and temperature at 17% moisture content and 150 rpm screw speed; (b) Screw speed and temperature at 17% moisture content and 10% FDDG level; (c) Moisture content and screw speed at 10% FDDG level and 120°C temperature

Torque increases with increase in FDDG level (Figure 6a) which also means that it decreases with increase in garbanzo flour. This is in agreement with findings of Bhattacharya and Prakash (1994). The torque during extrusion ranged between 9 and 19 N.m and high torque was associated with low screw speed. The effect of screw speed on torque also depends on the level of temperature. At high extrusion temperature, the response surface plot (Figure 6b) shows that torque value is almost constant with change in screw speed but at low extrusion temperature torque increased with decrease in screw speed. Filli et al. (2012) also reported decrease in torque with increase in screw speed and feed moisture during single screw extrusion of millet‐soybean mixture. Since the blends showed shear thinning behavior inside the extruder, the net torque required by the screw to convey the dough through the extruder decreased significantly. According to Guha, Ali, and Bhattacharya (1997), the decrease in the magnitude of torque with increase in screw speed can be explained by the reduced degree of fill in the extruder. Decrease in torque with increasing moisture content (Figure 6c) suggests more water is available for starch gelatinization resulting in reduction in apparent viscosity. Melt viscosity is low at high moisture contents and hence less torque will be required to work the material in the screw channels. Reduction in torque can be attributed to reduced friction in the extruder because of increase in feed moisture. This indicated that increasing moisture content or screw speed reduces the difficulty of processing. Similar results were found by Onwulata, Mulvaney, and Hsieh (1994) during twin screw extrusion of corn meal and by Chang and El‐Dash (2003) during extrusion of cassava.

## 4 CONCLUSIONS

The apparent viscosity, MFR, torque, and SME were shown to be significantly influenced by the extruder operating conditions. FDDG level and extrusion temperature significantly affected the apparent viscosity, torque and SME. Apparent viscosity increased with the increase in FDDG content. Higher feed moisture and higher extrusion temperature reduced the viscosity. With increase in the screw speed and feed moisture content the MFR also increased. Increasing the moisture of the blends and the extrusion temperature resulted in a decrease in torque. High SME was observed at high screw speed and low extrusion temperature.

## CONFLICT OF INTEREST

None declared.
